# Assessment of Glyphosate Impact on the Agrofood Ecosystem

**DOI:** 10.3390/plants10020405

**Published:** 2021-02-20

**Authors:** Yaxin Sang, Juan-Carlos Mejuto, Jianbo Xiao, Jesus Simal-Gandara

**Affiliations:** 1 College of Food Science and Technology, Hebei Agricultural University, Baoding 071001, China; sangyaxin@sina.com; 2 Department of Physical Chemistry, Faculty of Science, University of Vigo—Ourense Campus, E32004 Ourense, Spain; xmejuto@uvigo.es; 3 State Key Laboratory of Quality Research in Chinese Medicine, Institute of Chinese Medical Sciences, University of Macau, Taipa, Macau, China; 4 Nutrition and Bromatology Group, Department of Analytical and Food Chemistry, Faculty of Food Science and Technology, University of Vigo—Ourense Campus, E32004 Ourense, Spain

**Keywords:** glyphosate use, resistant weeds, AMPA, cancer risks, herbicide research

## Abstract

Agro-industries should adopt effective strategies to use agrochemicals such as glyphosate herbicides cautiously in order to protect public health. This entails careful testing and risk assessment of available choices, and also educating farmers and users with mitigation strategies in ecosystem protection and sustainable development. The key to success in this endeavour is using scientific research on biological pest control, organic farming and regulatory control, etc., for new developments in food production and safety, and for environmental protection. Education and research is of paramount importance for food and nutrition security in the shadow of climate change, and their consequences in food production and consumption safety and sustainability. This review, therefore, diagnoses on the use of glyphosate and the associated development of glyphosate-resistant weeds. It also deals with the risk assessment on human health of glyphosate formulations through environment and dietary exposures based on the impact of glyphosate and its metabolite AMPA—(aminomethyl)phosphonic acid—on water and food. All this to setup further conclusions and recommendations on the regulated use of glyphosate and how to mitigate the adverse effects.

## 1. Introduction

**Figure 1:**
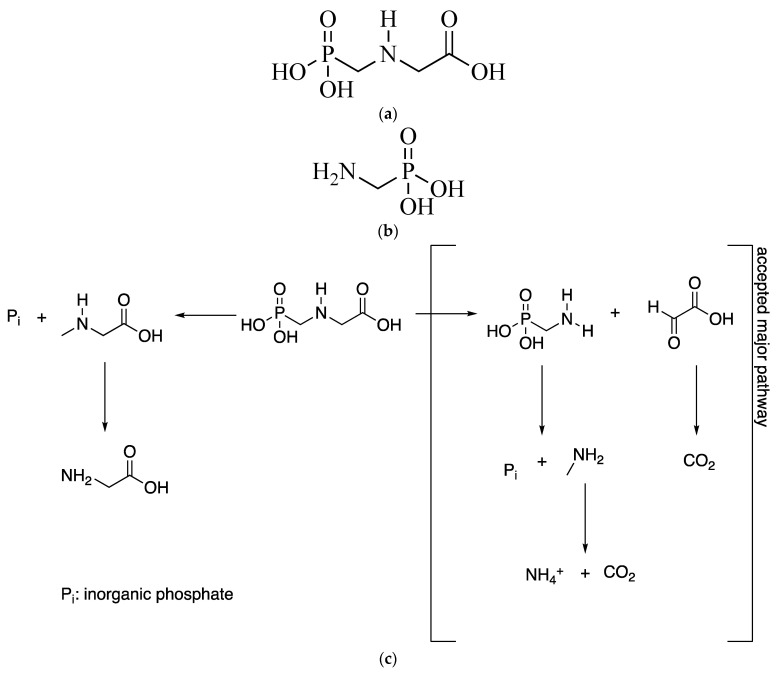
Structural formula of glyphosate (**a**) and AMPA –(aminomethyl)phosphonic acid– (**b**), together with degradation pathway for glyphosate in soil (**c**).

Glyphosate (*N*-phosphonomethylglycine; Figure 1a) is an aminophosphonate. This compound is typically used as a broad-spectrum herbicide and is absorbed by plant leaves. Glyphosate, discovered in the 1970s, was registered in more than 130 countries [1], and the use of glyphosate-based herbicides increased 100 times since then [2]. Genetically engineered herbicide-tolerant (GEHT) crops have considerably facilitated weed management in cotton, soybean, and maize [3,4,5]. However, they have also caused the emergence of glyphosate-resistant weed phenotypes [3,4,5,6,7,8]. The incorporation of additional herbicides into spraying programs [6,7] has caused herbicide per hectare on crops with GEHT varieties to escalate in this century [5,8,9]. This upward trend is expected to result in heavier environmental loads and increased human exposure to herbicides, including glyphosate and its main metabolite, aminomethylphosphonic acid (AMPA; Figure 1b), and to the adjuvants contained in its formulations. Weed management should face resistance before it happens [10]. It is key to promote changing crops or crop rotations against herbicide resistant (HR) weeds with effective herbicides [11]. Non-herbicidal alternatives (natural products, selective herbicides, mechanical controls, etc.) need to be added to satisfy the reduced efficacy of herbicides [12].

Figure 1c shows the degradation pathway for glyphosate in soil [13]. Although leaching of glyphosate is very unlikely due to its high soil sorption, depending on the type of soil, it can move to ground and surface waters through leaching and runoff [14]. Human exposure to urban sources of glyphosate should be considered too. Although some nonselective (broad spectrum) herbicides for both urban and home use in emerging countries contain glyphosate at low levels—and pose little risk of acute toxic exposure as a result [15]—those used in developing countries contain higher levels of this compound, or are mixed without official control. Dietary exposure in areas lacking residue information can be assessed from data for areas where glyphosate use and residues have been accurately determined [16].

The glyphosate mechanism as herbicidal involves inhibition of 5-enolpyruvylshikimate-3-phosphate synthase (EPSPS), which interferes with phenylalanine, tyrosine, and tryptophan synthesis. Unlike plants and some microorganisms, mammals have no EPSPS, which is in principle an advantage safety-wise [17]. However, glyphosate herbicides are highly controversial in toxicological and environmental terms. This review, therefore, diagnoses on the use of glyphosate and the associated development of glyphosate-resistant weeds. It also deals with the risk assessment on human health of glyphosate formulations through environment and dietary exposures based on the impact of glyphosate and its metabolite AMPA on water and food. All this to setup further conclusions and recommendations on the regulated use of glyphosate and how to mitigate the adverse effects in the below selected sections. The literature search was done following the guidelines included in the Preferred Reporting Items for Systematic Reviews and Meta-Analyses guide (PRISMA). Accordingly, a search was carried out on the following databases: Cochrane library, Embase, Medline, Cinahl, Scopus, Sci-Finder, and Web of Science. Scientific reports included in the study were obtained using the following search terms: “(glyphosate) AND (keywords of the different sections below)”. Common inclusion/exclusion criteria for references selection were based on publication date, mainly in the past 20 years, of both scientific-based papers and technical reports, with study designs, interests, and world overall coverage, applicable to all research questions below.

## 2. Glyphosate-Resistant Weeds

The international database on herbicide resistance [18] contains more than 510 studies. The best resistance mainframe is based on prevention and on detection with regular appraisal of herbicides-treated fields [19]. There are various methods to detect resistance with tests in the field and with bioassays in greenhouses and laboratories [20]; for example, hybridization between *A. palmeri* and *A. spinosus* occurred with frequencies in the field studies ranging from <0.01% to 0.4%, and 1.4% in greenhouse crosses [21]. Non-target-site resistance (NTSR) to herbicides in weeds can be conferred as a result of the alteration of one or more physiological processes, including herbicide absorption, translocation, sequestration, and metabolism. The mechanisms of NTSR are generally more complex to decipher than target-site resistance (TSR) and can impart cross-resistance to herbicides with different modes of action. Metabolism-based NTSR has been reported in many agriculturally important weeds, although reduced translocation and sequestration of herbicides has also been found in some weeds [22,23]. Crossed resistance is when the plant developed resistance to an herbicide, which permits to resist herbicides with the same action mode [24]. Multiple resistance is when a plant has one or several mechanisms of resistance to herbicides with distinct action modes. The selection pressure of an herbicide is then capable to select resistant plant biotypes depending on the herbicide treatment type, its formulation, application frequency, and the biological characteristics of the weed and the crop [25,26,27,28]. Examples of glyphosate-resistant weeds and their locations can be found in Table 1.

The problem is compounded by non-target site multiple resistances in grasses, as is the case of *Lolium rigidum* and *Alopecurus myosuroides* [12,26]. In addition, the growing expansion of multiple resistance to broadleaf weeds is bound to worsen things in the future. Managing non-target site resistance is difficult owing to the many unpredictable resistance patterns against which rotating herbicide sites of action may be ineffective. Herbicides are the main means for weed control in developed countries, but they should be used more sustainably [30]. This entails not only using improved herbicide mixtures and rotations, but still adopting intensive integrated weed management programs including effective mechanical and cultural strategies [31]. A pressing need therefore exists for economic incentives to the search for new, safer, and more effective herbicides [4].

Developing herbicide-resistance crop traits may grant the use of old herbicides in new ways through tailored mixtures efficiently avoiding multiple resistance [32,33]. Indeed, the use of different genetic engineering techniques as RNA interference (RNAi) [3,32,34,35,36,37,38], chimeric RNA/DNA oligonucleotides [39], and gene-editing techniques (GM), such as CRISPR/Cas9 or CRISPR/Cpf1 [40,41,42,43,44,45,46,47,48] technology, might be useful for this purpose. The best approach to prevent resistant weeds is to use a combined weed management, and herbicides will likely be partly replaced with new technologies such as, among others, research on crop allelopathy [49,50,51,52,53,54,55,56] and engineering of microbial control agents [57,58,59,60,61]. Progress in these technologies is expected to allow methods for weed control to be used in an integrated manner with the aim of maximizing diversity in weed control and minimizing resistance. Applying evolutionary principles to agricultural settings is essential to properly understand the system-wide effects of herbicide selection intensity [62]. Although the main driver of herbicide resistance is the selection pressure of management, further knowledge of the scientific bases of herbicide resistance at the genetic and cellular levels needs to be developed [63,64,65,66,67]. The causes and dynamics of resistance expansion might be elucidated by assessing the flexibility of certain alleles involved in herbicide resistance [68]. The capability of resistant weeds to prevail, replicate, and selectively infest habitats depends on the degree of vigour of the particular resistant gene [69]. The effects of the environment on resistant plants in cropping conditions could thus reduce the heritability and frequencies of resistance alleles with time [70].

Research in this field should also address the effects of climate change on the expansion of herbicide resistance. According to Renton [71], spatially computational models could be of help in this context by providing powerful indicators on how genetics, plant biology, population structure, environmental conditions, and management strategies connect to shape weed resistance dynamics. The use of scarcely diverse practices drove to a fast increment in multiple-resistance weeds with upgraded abilities for herbicide metabolism worldwide [72]. Elucidating herbicide metabolic pathways could help re-classify and re-rank the risks of herbicide resistance and hence enable the adoption of more effective herbicide rotations, such as those based on both site of action and metabolic pathway. Processes associated with climatic changes, such as elevated temperatures, can strongly affect weed control efficiency. For example, responses of several grass weed populations to herbicides that inhibit acetyl-CoA carboxylase (ACCase) were examined under different temperature regimes [73].

### Mitigation Strategies

Weed resistance management has three planks: rotate modes of action to reduce selection pressure, incorporate non-chemical practices and control weed seed set, together with asexual reproduction (rhizomes, stolons, etc.). Effective weed control needs to discern weeds biology, prevents with weed seed production, plant into weed-free fields, grow weed-free seed, and inspect lands regularly. There is also a need to adopt numerous herbicide action mechanisms active versus damaging weeds, spread herbicide estimate at selected weed extents, and highlight growing conditions that put an end to weeds by crop competition. Further, it is useful to practice mechanical and biological executive strategies, avoid field-to-field and within-field migration of weed vegetative propagules, regulate weed seed to avoid a reinforcement of the weed seed-stock, and preclude an invasion of weeds into land by controlling ground boundaries. All these diverse approaches to managing herbicide resistance need to be incorporated into weed management [74,75,76,77,78].

This will be beneficial in managing resistance in the long term, together with mathematical simulations proving that mixtures magnify herbicide efficacy, choice array of soil-applied herbicides, and postpone herbicide resistance growth in weeds. It shows than extension efforts rotating herbicide mixtures give vision to guide the progression of weed resistance [69]. Multiple modes of action (MOAs) for weed control are important for managing herbicide resistance and enabling no-till farming practices that help to sequester greenhouse gases, but discovering new herbicide MOAs has been a challenge for the industry [76].

According to the International Assessment of Agricultural Knowledge, Science and Technology for Development [79] (IAASTD, 2008), agricultural development has focused on increasing farm-level yield, more than on consolidating effects on biodiversity and the liaison of agriculture with climate change. Increased attention needs to be directed to build up soil fertility and to sustain agricultural production, with a focus also on protection of biodiversity. Agro-ecology refers to treating agricultural ecosystems as ecosystems, and can enable a successful transition to more sustainable farming and food systems [80].

Moreover, in recent decades, studies were performed looking for alternatives to glyphosate. There is a rise in efficacy tests using different natural (or even modified) allelo-chemicals obtained from essential oils for pest-control [81,82,83,84,85,86,87,88,89,90,91,92,93,94,95,96], as well as in their use as herbicides [97,98,99,100,101,102,103,104,105,106,107,108,109,110,111,112].

## 3. Impact of Glyphosate and Its Metabolite AMPA on Water Streams

Glyphosate residues raised perception of its adverse effects on human health, soil, and aquatic ecosystems [113]. Some microorganisms in soil and water can degrade this compound [114]. The major metabolite of glyphosate is aminomethylphosphonic acid (AMPA; Table 2).

Glyphosate can flow throughout soil, and reach surface and ground waters [14,115,116,117]. Although sorption and degradation are affected by many factors that might be expected to affect glyphosate mobility in soils, glyphosate leaching seems mainly determined by soil structure and rainfall. Limited leaching has been observed in non-structured sandy soils, while subsurface leaching to drainage systems was observed in a structured soil with preferential flow in macropores, but only when high rainfall followed glyphosate application [14]. The time needed for glyphosate in river water to be eliminated by 50% (i.e., DT_50_) has been found to range from 13.8 to 301 days, which is suggestive of moderate to high persistence [118]. Rivers are influential environments with a fundamental action in xenobiotic mitigation [119]. Thus, river water usually transports nutrients, organic matter, and pollutants that can severely constrain microbial growth [120]. Biofilms, which are network structures with a wide range of microbes, contribute to transform xenobiotics through co-metabolism and mineralization. Glyphosate can be utilized to obtain phosphorus by microorganisms such as bacteria and fungi in biofilms [121,122]. The compound is cleaved through the carbon–phosphorus lyase (C-P lyase) route, which comprises consecutive enzyme-catalysed reactions including phosphonate activation and C–P bond break [123]. The genes enciphering the enzymes are amassed into the phosphonate operon, which occurs widely amongst bacteria. However, the effects of phosphorus on glyphosate degradation have been investigated in segregated microbial strains [124] rather than in natural biofilms [125].

AMPA is a metabolite from glyphosate and from aminopolyphosphonate, which is applied in detergents, flame-retardants, and anticorrosive products [126]. As found by meta-analysis [127], glyphosate and AMPA are concomitantly present in water, with levels evolving in the same way at positively correlated concentrations, since glyphosate can be rapidly converted to AMPA. Battaglin et al. [128] detected glyphosate, but no AMPA in 2.3% of 3732 water and sediment specimens. Moreover, Struger et al. [129] found the parent compound and its metabolite to co-occur at probability *p* value = 0.76 in Canadian rivers. The concomitance of glyphosate and AMPA in groundwater suggests partial mineralization of the former under the influence of anthropic activities or environmental conditions. Glyphosate is converted into AMPA largely in eutrophic water than it is in P-poor water [127]. Glyphosate and AMPA have been identified at oligotrophic sites and found to be completely absent from upstream communities under low glyphosate–low phosphorus conditions. Glyphosate can have from low to high persistence in soils with aerobic conditions (DT_50_ 2.8–500.3 days), and high persistence in anaerobic soils (DT_50_ 135–1000 days) [118]. Glyphosate persistence in water sediments is moderate to high (DT_50_ 13.82–301 days) [118]. Phosphorous from glyphosate and AMPA is very low accounting for <0.17% of total P. The degree of contamination of surface water with glyphosate depends largely on two factors, namely: (a) herbicide level, affected by soil biodegradation and sorption; and (b) phosphorus availability in the water, reducing its degradation. This should be considered in the evaluation of the environmental risks of glyphosate and AMPA present in surface waters [121,127,130,131,132,133,134,135,136,137].

As some people might use surface-water for drinking and preparation of food, it was assumed that it was consumed untreated. Based on the median (0.03 µg/L) and 99th centile (302 µg/L) concentrations found in surface-water, oral doses were 0.00000086 and 0.0043 mg/kg b.m./d. These exposures are considerably less than the acceptable daily intakes (ADIs) and present de minimis risk [138]. Glyphosate can be bound to divalent and trivalent cations in the soil and water, but there is risk to aquatic life joined to the residues of free available glyphosate in water [139]. The hazard quotients (HQs) obtained in sediments and the repercussion over benthic creatures were of 1.4–6.7, proposing risk for sediment dwellers. Annett et al. [140] and Thompson et al. [141] estimate HQs higher than one for fish and for aquatic microorganisms, together with invertebrates and amphibians. The HQs for AMPA advice it is not threatening. Glyphosate seems to be critical where intensive agriculture is practiced, since it involves increased use of fertilizers, plant growth regulators, and pesticides and mechanised agriculture [130,139,140,142].

### Mitigation Strategies

Glyphosate residues are linked to its applications calendar and soil inputs; thus, the need to reduce overspray, but also to focus on its relative mobility/persistence [143]. Residual levels in sediments and water streams may be diluted with the upper part of the basin devoted to woodland, because it is not used in woodlands, and woodlands themselves help to reduce and prevent diffuse pollution [144]. There is a need to develop sediment quality guidelines for such contaminants [145]. The actions to restrict them in surface and groundwater below current water quality standards can be at the exploitation level (collection and treatment of wash water for sprayers or engines…), at field plot level (reduction of the dose, application date according to the weather…), and at catchment level (vegetated buffer strips, orientation of the crop rows…). Artificial wetlands have to be implemented in addition to local action, such as a pesticide reduction plan [146]. The most commonly used mitigation techniques to prevent pesticide input into water bodies include edge-of-field and riparian buffer strips, vegetated ditches, and constructed wetlands. It has been identified that removal of pesticides is highly variable, and generally increases with increasing value of K_OC_, but the relationship is not strong [147]. All these undertakings should help with the mandatory regulations for Sustainable Use of Pesticides Directive, which include each EU member state having a National Action Plan on the reduction in the use of pesticides, buffer zones, prevention of contamination of watercourses, etc.

## 4. Glyphosate-Based Herbicides and Cancer Risks

The mechanisms of action of glyphosate herbicides involve endocrine or microbiome disruption (Table 3).

International regulatory agencies typically classify substances according to their dose–response relationships, thereby overlooking non-monotonic carcinogenic issues in glyphosate. Usually, toxicological data were obtained with an incomplete judgement of the outcome of hormone imitation and the microbiome. Some agencies, including US EPA—United States Environmental Protection Agency, EFSA—European Food Safety Authority, and Canada’s Pest Management Regulatory Agency, are reviewing studies on glyphosate’s effects on human health and species at risk to protect farm workers, food safety, and endangered species. The present decision for the parts of the assessment that are complete, which stands until the next review, shows that can still be recommended when used according to instructions on the label. In March 2015, IARC -International Agency for Research on Cancer—categorized glyphosate as “probably carcinogenic to humans” (Group 2A) based mainly on research demonstrating that ‘there was limited evidence of carcinogenicity in humans’, mostly from agricultural workers, but also concluded that there was ‘sufficient evidence of carcinogenicity in experimental animals’ [163,164,165,166,167]. IARC also concluded that there was evidences for genotoxicity, both for active ingredient and formulations. According to De Roos et al. [168] and further [169], the main conclusion is that “The most reliable approach will be to reanalyse the data after more cases accumulate, both to assess whether the association with myeloma persists and to further evaluate confounding and selection bias using a larger case group to support analyses”. With the same prospective cohort study, Andreotti et al. [170] concluded that there was some evidence of increased risk of acute myeloid leukaemia (AML) among the highest exposed group that requires confirmation. Their effects caused by disturbance of cell–cycle management might also be important for cancer and non-cancer health outcomes [171,172].

There has been a strong controversy over the use of this herbicide and the detection of potential toxic consequences of pure glyphosate itself and glyphosate-based herbicide ingredients that might have a synergistic effect, such as the surfactants used. The surfactants in glyphosate formulations (especially polyoxyethylene, POE-15) are major contributors to DNA damage caused by glyphosate-based herbicides (GBH). Such surfactants have demonstrated to alter mitochondrial function [173] and are also deleterious to human embryonic and placental cells at concentrations around 1 ppm [174]. Richard et al. [175] found Roundup formulation with surfactants to be more than double active than glyphosate alone at producing lethal danger in human placental cells. Moreover, Guilherme et al. [176] found increased numbers of double-strand breaks (DSB) with the comet assay and micronucleus (MN) lesions in eels after exposure to environmental levels of Roundup (0.05 ppm). Therefore, there is a need to regulate the use of GBH in mixtures. In this regards, it was found that glyphosate and its overall formula show genotoxicity in vivo and in vitro [177]. Polyethoxylated tallow amine (POEA) and other surfactants were also found to be toxic [178]. EU prohibited POEAs in glyphosate formulas, but EPA permitted it at up to 25% (w/w). IARC [179] evidenced that glyphosate formulas produce non-Hodgkin lymphoma (NHL), according to epidemiological research. EPA focused on the Agricultural Health Study [170], finding no relationship of glyphosate with NHL incidence in U.S. applicators. Leon et al. [180] obtained a meta-hazard ratio of 1.36 between diffuse large B-cell lymphoma and glyphosate. Zhang et al. [181] detected that most exposed users had a 41% higher risk of NHL. EPA’s Office of Research and Development (ORD) concluded that glyphosate is “likely to be carcinogenic” or “suggestive of carcinogenicity” [182,183].

The results depend on the particular cell, application, and design specifications. Thus, the overall formula exhibits a linear dose-dependent response indicating that toxicity from the adjuvants is monotonic [184]. Gasnier et al. [185] found glyphosate at concentrations below 0.05% to have a non-linear effect on oestrogen receptor-reporter transfected HepG2 cells and the full formulation to linearly reduce androgen receptor-induced transcription with low concentrations. Testosterone-producing Leydig cells afford an alternative model for endocrine disturbance in vivo and ex vivo [148]. Walsh et al. [186] detected disturbed progesterone yield, but only with the full formula, which altered puberty progression and reduced serum testosterone in pre-pubertal Wistar rats at 5 mg kg^−1^ day^−1^. Some authors [187,188,189] recognized the non-monotonicity of glyphosate itself on a human hormone-reliant cell line of breast cancer. They found the effect to be propitiated by the oestrogen feedback and hindered by inclusion of an oestrogen receptor antagonist. Armiliato et al. [190] reported elevated expression of steroidogenic factor-1 and oocyte rise in zebra fish in the microgram-per-litre range in water. However, no significant association with endocrine disruption was found in trout. Glyphosate did not elevate vitellogenin plasma concentrations in young rainbow trout [191]. Gandhi et al. [192] found environmental concentrations of glyphosate in water to alter behaviours such as movement frequency in larval amphibians. If this was the result of a non-monotonic mechanism, then, even very low doses may have some effect on the nervous system. Thus, low levels of glyphosate—even those below regulatory limits—may boost human carcinogenesis through endocrine mimicry.

AMPA is also seemingly genotoxic. Guilherme et al. [193] found 11.8 μg L^−1^ concentrations of the glyphosate metabolite to induce DSB in an eel model. In addition, Mañas et al. [194] found AMPA to induce cracks at 2.5 mM in human lymphocyte cultures and in mice. Calculations of total exposure to this degradation product should therefore include residues potentially present in organisms and the environment. Some animal cancer works were proving increased risks of hemangiosarcoma, renal tubule carcinoma, and pancreatic cell islet adenoma, together with skin tumour build up in a mouse model [165]. If both the parent molecule and its metabolite are carcinogenic, then the risk cannot be accurately assessed with the standard Paracelsian dose-response model.

The exposure scenario assessment reflects short-term incidental oral exposure to glyphosate-treated park areas (post-application exposure). The short-term assessment is protective of intermediate-term exposure, and the life-stages selected for aggregate risk assessment are considered protective for the exposures and risks for any other potentially exposed life-stage, since the resulting margins of exposure (MOE), which are Incidental oral NOAEL (No observed adverse effect level)/Residential post-application total exposure, are of 2,200,000 for adults, and of 640 for children 1 to <2 years old [163]. For a chemical substance with health thresholds (i.e., not genotoxic and not carcinogenic), a MOE ≥ 100 is generally considered to be protective. Instead, for genotoxic and carcinogenic compounds, in general a MOE ≥ 10,000 is considered to be protective.

### Mitigation Strategies

Glyphosate’s use is four times higher than atrazine (the second pesticide in the list) [195]. For this reason, the main mitigation strategy is the control of its use. There is typically a time lag of decades between exposure to a carcinogen and elevated cancer rates, and glyphosate use has skyrocketed over the past 10–15 years, the full effects of glyphosate’s rising use remain to be discovered [196].

## 5. Risk Assessment of Glyphosate through Environment and Dietary Exposures

Pesticides, such as glyphosate, are evaluated periodically for changes. In the case of glyphosate, there is a strong controversy with the results obtained by different authors and the conclusions of different agencies. These contradictions have led glyphosate use to being banned or strictly regulated in some countries. Based on dietary risk assessment [197], glyphosate’s NOAEL is 100 mg/kg·day. It is 1114 times higher than the exposure for the US population, and 438 times higher than that of kids between 1 and 2 years [163]. Acquavella et al. [198,199,200] found a maximum systemic dose for farmers of 0.004 mg/kg. McGuire et al. [201] monitored glyphosate in the urine of breastfeeding women. It was found that only 20% of dietary glyphosate is available, and most of it is excreted in the urine [138,202]. Stephenson and Harris [203,204], considering food processing on glyphosate residues, reduced estimated exposures by 67-fold. Drinking water exposures can be estimated with models based on physical properties of the pesticide, its use, and environmental variables as soil type and rain [205]. Glyphosate is detected in the urine of farm and non-farm family members, kids included, with analogue exposure [206,207]. Glyphosate was also found in human blood [208]. EPA’s safe maximum of glyphosate exposure is six times that of Europe’s [209]. EPA’s estimation of children exposure to glyphosate is higher than the maximum level suggested [210,211]. All of the environmental exposure studies at Connolly et al. [212] had mean/median levels that were 2% of the ADI or less, and the maximum concentrations found in these studies were all less than 6%. One study on residential exposures showed median and maximum values that were 49% and 53% of the ADI, respectively, while another study reported a maximum value was 87% of EFSA’s ADI; these studies with the highest percentage compared to the ADI are studies that were outside Europe and involved aerial spraying.

### Mitigation Strategies

The key is post-approval monitoring [213]. Such control will track tendencies, identify inflection points, and measure the efficacy of past risk-mitigation assays. Many of the inert ingredients in formulated pesticides are themselves toxic [197,214,215], or help the active ingredient to endure in biological systems. It could be simple to estimate a 5-year rolling average number of herbicides kill units to bear a crop to harvest [216]. If the kill unit begins to slope upward, a new pest management action is necessary. Risks could be reduced by commanding identical pre-harvest intervals on herbicides leading to higher residues, similar number of application rates, compulsory resistance management, and decreased tolerances to prohibit applications. Plans are necessary for these strategies to become real [211,217,218].

## 6. Challenges and Opportunities for Herbicide Research and Development

The expected growth in the global population will inevitably have to be met by increasing food production. Although the arable land area seemingly remains stable [219], the increasing loss by effect of urbanization and climate change must be considered. Historically, cities have grown in places where good arable land was available [220]. This forced production sectors to increase their output through, for example, efficient weed control. The decreasing variety of effective herbicides and modes of action has had an adverse impact on plant diversity and is hampering sustainable weed management [221]. The introduction of new weed species through international transport of goods is posing additional problems. In addition, the increasingly frequent occurrence of environmental extremes [222] may affect weeds disparately and alter crop responses. As shown by the giant hogweed (*Heracleum mantegazzianum* Sommier & Levier), invasive weeds can pose serious problems to European authorities [223].

Screenings for structure–activity relationships and virtual screening technologies [97] are helpful to select compounds with diverse structures but identical performance. Active principles with new modes of action should be well tolerated by crops, easy to apply, cost-effective, and amenable to regulation [98]. The agro-research industry is aiming to find effective herbicidal solutions to help sustain weed management diversity and crop production.

## 7. Conclusions and Recommendations on the Regulated Use of Glyphosate

The aim of the Farm to Fork strategy and the European Green Deal is to implement sustainable and environmentally friendly policies, in particular in agriculture, and this encompasses the protection of human health and biodiversity. Therefore, herbicides in general, and glyphosate in particular, are recovering our attention.

Glyphosate is a non-selective herbicide commonly used in croplands, urban areas, homes, and gardens. We can be exposed to this compound and its degradation components through the foods and the environment. In response to reclassification of glyphosate in Group 2A (probably carcinogenic to humans), regulations that are more stringent were implemented to set the maximum levels for glyphosate in livestock and poultry food products including meat, milk and eggs, but also in different crops.

Biological pest control, organic cropping, and regulative management help to reduce glyphosate use. Agro-ecology has drawn increasing interest and, according to many stakeholders, represents a strategic approach that can enable a successful transition to more sustainable farming and food systems. The policy in favour of agro-ecology would be exceptional, because it addresses all the levers needed to promote the agro-ecological transition, from production to consumption, by way of a transformation of the systems of education, research and development for achieving dietary security in the ever-lengthening shadow of climate change, bringing us thus closer to the realization of the plan in the 2030 Agenda for Sustainable Development and its 17 Sustainable Development Goals.

The potential carcinogenicity, massive use, and increasing presence of glyphosate residues in drinking water sources should lead regulatory agencies to take actions such as the following to protect human health: (a) Making trace level analyses in food and water mandatory; (b) re-assessing acceptable daily glyphosate intake levels; and (c) adding glyphosate to the water quality standards for drinking water sources. In this way, under the Sustainable Use of Pesticide Directives, each member state in the EU must have a national action plan that requires ‘quantitative objectives, targets, measures and timetables to reduce risks and impacts of pesticide use on human health and the environment’ which also includes measuring for residues. Secondly, they state a requirement for re-assessing the ADI values, but this already occurs when chemicals are re-evaluated to renew their licence to be on the European Market.

Environmental loads and exposure to glyphosate, AMPA, and formulation adjuvants continue to increase. Urban use of glyphosate in emerging countries is also a key issue. The European Food Safety Authority (EFSA) succeeded in identifying the potentially deleterious consequences of glyphosate on untargeted wild terrestrial vertebrates [118] in different scenarios such as crops pre-planting, post-planting and pre-emergence, cereals and oilseeds pre-harvest, and orchard crops and grapes.

There has been a strong controversy over the use of this herbicide and the detection of its residues in various foodstuffs. In 2013, the German Institute for Risk Assessment (BfR) conducted a comprehensive study concluding that classifying glyphosate as a carcinogen was unwarranted. This conclusion was reinforced by the EFSA in 2014 and 2015 by deeming it unlikely for glyphosate to pose serious risks to humans, in line with EPA’s statements of 1993, 2015, and 2016 that glyphosate was probably not carcinogenic. However, some of the scientific community refuted these claims, some agreed, and some just stated that further research and work is required for this chemical, as it was already supported in previous sections. This is because no information about potential conflicts of interest of the authors of the reports was revealed or even that they were verbatim copies of previous studies produced by the multinational Monsanto, currently owned by Bayer. In that way, the public was served with controversy and serious doubts about potential prevarication on the part of the interested stakeholders. On top of that, in 2015, the WHO agency IARC classified glyphosate as probably carcinogenic to humans (Group 2A) after analysis of publish scientific papers that associated to some cases of lymphomas (vide supra). These contradictions have led glyphosate use to being banned or strictly regulated in some countries. There were other issues with these evaluations, such as the IARC was evaluating for hazard, while the European Food Safety Authority (EFSA) and European Chemicals Agency (ECHA) were evaluating for risk. The difference between a hazard and risk assessment is the inclusion of probable exposure levels expected. Thus, the massive use of glyphosate and the expansion of glyphosate-resistant transgenic plants has allowed extensive agricultural production to be increased with substantially reduced costs. In this respect, the agrochemical can help fulfil the Millennium Goals and the 2030 Agenda by allowing adequate amounts of food to be supplied to a growing population. Moreover, it is clear that banning glyphosate use would increase tillage—and agricultural production—costs in some European Union countries. On a market with total price freedom, and under control of logistics and distribution multinationals, farmers are forced to keep prices low in order to compete with non-EU producers, many of whom can still use glyphosate virtually freely. As a result, EU farmers are having to sell their products at prices below their production costs, thus causing strong economic stress in European agriculture. In any case, there is a shadow of doubt about the safety of glyphosate as an herbicide, so appropriate measures should be taken in this respect to protect public health, and the environment. Furthermore, a need exists to avoid unfair competition from producers in countries where different regulatory values are in force.

It is key to finance epidemiological, biomonitoring, and toxicological research on endocrinology to verify if the consequences of glyphosate are mediated by endocrine disturbance, for example. That is the main conclusion that we want to make clear, recommending further follow-up to clarify this matter.

## Figures and Tables

**Table 1:** Example of glyphosate-resistant weeds and their locations, extended from reference [29].

Weed	Location
*Amaranthus palmeri*	United States
*Amaranthus tuberculatus*	United States
*Ambrosia artemissifolia*	United States
*Ambrosia trifida*	United States
*Conyza bonariensis*	United States, Brazil, Argentina
*Conyza canadensis*	United States
*Euphorbia heterophylla*	Brazil
*Lolium perenne*	United States, Brazil, Australia
*Sorghum halepense*	United States, Argentina

**Table 2:** Properties of glyphosate and its metabolite AMPA.

Common Name	Glyphosate	AMPA
Chemical name	*N*-phosphonomethylglycine	Aminomethylphosphonic acid
CAS number	1071-83-6	1066-51-9
Molecular formula	C_3_H_8_NO_5_P	CH_6_NO_3_P
Exact mass	169.01 g mol^−1^	111.01 g mol^−1^
Vapour pressure (25 °C)	1.31 × 10^−5^ Pa	8.44 × 10^−4^ Pa
Henry’s law volatility constant (25 °C)	2.1 × 10^−7^ Pa m^3^ mol^−1^	2.6 × 10^−3^ Pa m^3^ mol^−1^
Solubility in water (20 °C)	10.5 g L^−1^	1467 g L^−1^
Partition coefficient (log P_ow_) (20 °C)	−3.2	−1.6
Solid/water distribution coefficient (*K*_d_)	5.3–900 L kg^−1^	15–1554 L kg^−1^
Soil organic carbon normalized adsorption coefficient (*K*_oc_)	884–60,000 L kg^−1^	1160–24,800 L kg^−1^
Half-life (DT_50_) in soil	1–197 days	23–958 days
DT_90_ in soil	40–280 days	Unknown

**Table 3:** Some recent studies of glyphosate-based formulations’ toxicity involving various indicators such as organisms that can be affected and the main results at tested concentrations.

Herbicide Formulation	Test-Organism	Endpoint	Results	Tested Concentrations	Ref
Glyphosate	*Geotrichum candidum,* *Lactococcus lactis* subsp. *Cremoris;* *Lactobacillus* *delbrueckii* subsp. *bulgaricus*	Microbial growth assay	Inhibition of microbial growth by the commercial product Roundup; microbiocidal effect at concentrations below those recommended for agricultural use of the commercial product Roundup; no significant toxicity of the active ingredient (glyphosate) on any of the microorganisms	0.1, 1, 10, 100, 1000, 10,000 ppm	[148]
Glyphosate	Tadpoles of wood frog (*Rana sylvatica* or *Sylvaticus lithobates*), leopard frog (*Rana pipiens* or L.) and American toad (*Bufo americanus* or *Anaxyrus americanus*)	Acute toxicity assay	Significant induction of morphological alterations in tadpoles of the three species; exposure to glyphosate altered tadpole tail size in wood and leopard frogs at all tested concentrations	0, 1, 2, or 3 mg acid equivalents [a.e.]/L of Roundup Original MAX	[149]
Glyphosate	Roots from the smooth hawksbeard (*Crepis capillaris* L.); polychromatic erythrocytes of the bone marrow of C57BL rat	Chromosome aberration assay; micronucleus assay	No induction of genotoxic and/or mutagenic effects on any of the species	*Crepis capillaris*: 0.05, 0.1, 0.5, 1%; erythrocytes: doses inferior to half the LD_50_ (1080 mg/Kg)	[150]
Glyphosate	Female Wilstar rats	Acute toxicity assay; teratogenicity assay	High mortality index of females treated with the highest concentration of the commercial product Roundup; increased dose–response of foetal skeletal alterations	500, 750, 1000 mg kg^−1^	[151]
Glyphosate	Human lymphocytes	Comet assay; FISH; lipid peroxidation assay–TBARS	Significantly increased DNA migration at 580 μg mL^−1^; significantly increased comet tail intensity at 92.8 μg mL^−1^; increased DNA damage in the presence of S9; increased frequency of micronuclei, nuclear buds and nucleoplasmic bridges, without S9; significantly increased nuclear instability at the highest concentration tested with S9; significantly increased dose–response of TBARS levels	0.5, 2.91, 3.5, 92.8, 580 μg mL^−1^	[152]
Glyphosate; 2,4-D	Algae and 25 species of aquatic animals	Acute toxicity assay	No reduction in periphyton biomass by either herbicide; no strong impact of 2,4-D on the aquatic community; strong impact of glyphosate on the aquatic community (significantly decreased species richness)	0, 1, 2, or 3 mg acid equivalents [a.e.]/L of Roundup© Original MAX	[149]
Glyphosate/As As/Cu	Soil nematode *Caenorhabditis elegans*	Heat Shock Protein Response, Reproduction and Locomotory behaviour (head thrashing)	Responses in locomotory behaviour (head thrashing), reproduction, and heat shock protein expression had been observed.	Sublethal 24-h exposures of 1/1000, 1/100 and 1/10 of the LC50	[153]
Glyphosate; Terbuthylazine	Human lymphocytes	Cytome FISH	Glyphosate concentrations above 3.5 μg mL^−1^ increased the frequencies of micronuclei, nuclear buds and nucleoplasmic bridges in treated cells without inducing centromeric signals; terbuthylazine at concentrations above 0.008 μg mL^−1^ increased the frequency of micronuclei hybridized with centromeric probe and of nuclear buds with centromeric signals in the presence of S9	0.5, 2.91, 3.50, 92.8, 580 μg mL^−1^ (glyphosate); 0.00058, 0.0008, 0.008, 25, 156,5 μg mL^−1^ (terbuthylazine)	[154]
Glyphosate	Earthworms *Pontoscolex corethrurus* *Amynthas corticis*	Toxicity assay	Coffee plantations with regular applications of Glyphosate over the preceding 22 years. Control plantations had received no herbicides over the preceding 7 years. The earthworm species found in plots with no treatment were *Pontoscolex corethrurus* (99%) and *Amynthas corticis* (1%), while *A. corticis* was absent in plots that had been treated.	Manufacturer’s recommendations	[155]
Glyphosate	*Allium cepa*	Cytotoxic evaluation Cytogenotoxic effects	Exposure to glyphosate of *A. cepa* meristematic cells induces diverse types of chromosomal anomalies in demonstrates that it has a highly cytogenotoxic effect for any of the concentrations used.	5, 10, 15, 25, 30 mg L^−1^	[156]
Glyphosate, alkylphenolpolyglycol ether	Neotropical fish *Piaractus mesopotamicus*, *Phallocerus caudimaculatus*, *Hyphessobrycon eques*, *Brachydanio rerio*	Toxicity assay, Histopathological effects	The histopathological effects caused by glyphosate exposure on gills, liver, and kidneys are reversible, except for the liver necrosis on *P. caudimaculatus. H. eques, P. caudimaculatus*, and *P. mesopotamicus* present great potential to be used as standard organisms for herbicides monitoring and the use of glyphosate without surfactant addition is enough to cause histological alterations on *H. eques* and *P. caudimaculatus*	Manufacturer’s recommendations Rodeo© Rodeo©+0.5%Aterbane©BR Rodeo©+1.0%Aterbane©BR	[157]
Glyphosate/P	*Capsicum annuum* Inoculated and non-inoculated with *Glomus mosseae* or *Glomus intraradices*	Accumulation of shikimic acid in mycorrhized *Capsicum annuum* L.	Remobilization of glyphosate residues in the soil by the addition of phosphate should be considered a serious problem for crops in treated soils. The mycorrhization increases the pepper plant’s tolerance to high glyphosate concentration in the substrate, and may allow support to this stress condition	Manufacturer’s recommendations RoundUp©	[158]
Glyphosate	*Misgurnus anguillicaudatus*	Toxic assay	Glyphosate represent a potential risk to loach through inhibiting proliferation of diploid and triploid cell lines and induces micronuclei and apoptosis.	80, 240, 400, 560, 720, 880, 1040 mg/L	[159]
Glyphosate	*Pouteria torta*	Changes in the biological performance	In response to glyphosate, *P. torta* exhibited reductions in photosynthesis and chloroplastid pigment content, as well as accumulation of shikimic acid and the occurrence of chlorosis and necrosis. These changes demonstrate use as a bioindicator of this herbicide.	25, 50, 100, 200, 400, 800, 1200 g a.e. ha^−1^	[160]
Glyphosate	*Crassostrea gigas*	Embrio-larval development and metamorphosis	Embryo-larval development of *C. gigas* was more sensitive to glyphosate-based herbicides compared to various endpoints studied in regulatory model organisms, and embryos and D-shaped larvae were more sensitive compared to pediveliger larvae.	0.1 to 100,000 μg L^−1^ RoundUp©	[161]
Atrazine, Glyphosate	*Biophalaria glabrata*	Cytotoxic assay	Results indicated that those atrazine and glyphosate herbicides may be considered to be highly genotoxicant agents	-	[162]

## Data Availability

Not applicable.
